# Access to Health Care for Migrants Along the Mexico-United States Border: Applying a Framework to Assess Barriers to Care in Mexico

**DOI:** 10.3389/fpubh.2022.921417

**Published:** 2022-07-14

**Authors:** César Infante, Isabel Vieitez-Martinez, César Rodríguez-Chávez, Gustavo Nápoles, Silvana Larrea-Schiavon, Ietza Bojorquez

**Affiliations:** ^1^Center for Health Systems Research, Instituto Nacional de Salud Pública, Cuernavaca, Mexico; ^2^Population Council México, Ciudad de Mexico, Mexico; ^3^Department of Population Studies, El Colegio de la Frontera Norte, Tijuana, Mexico

**Keywords:** health services access, migrants, health systems, accessibility, implementation gap, Mexico

## Abstract

**Background:**

Migrants in Mexico are entitled to care at all levels, independently of their migration status. However, previous studies show that access to care is difficult for this population. As the movement of in-transit migrants and asylum seekers has been interrupted at the Mexico-United States border by migration policies such as the “Remain in Mexico” program, and by border closures due to the COVID-19 pandemic, the Mexican health system has the challenge of providing them with health care. Levesque et al.'s framework, according to which access occurs at the interface of health system characteristics and potential users' abilities to interact with it, is a useful theoretical tool to analyze the barriers faced by migrants.

**Objective:**

The objective of this article is to analyze the barriers to access the public Mexican health system, encountered by migrants in cities in Mexican states at the Mexico-United States border during the COVID-19 pandemic.

**Methods:**

Data came from a multiple case study of the response of migrant shelters to health care needs during the COVID-19 pandemic. The study consisted of a non-probability survey of migrants with a recent health need, and interviews with persons working in civil society organizations providing services to migrants, governmental actors involved in the response to migration, and academics with expertise in the subject. We analyzed the quantitative and qualitative results according to Levesque et al.'s framework.

**Results:**

36/189 migrants surveyed had sought health care in a public service. The main limitations to access were in the availability and accommodation dimension (administrative barriers decreasing migrants' ability to reach the system), and the affordability dimension (out-of-pocket costs limiting migrants' ability to pay). Civil society organizations were a major source of social support, helping migrants overcome some of the barriers identified.

**Conclusions:**

While Mexico's health regulations are inclusive of migrants, in practice there are major barriers to access public health services, which might inhibit migrants from seeking those services. In order to comply with its commitment to guarantee the right to health of all persons, the Mexican health authorities should address the implementation gap between an inclusive policy, and the barriers to access that still remain.

## Introduction

Globally, migrants face multiple barriers in accessing health services, which go beyond those of the local, non-migrant population. These include legal barriers such as the exclusion of irregular migrants from publicly funded health care systems, the cost and availability of services, limited information on how to access services, language and cultural differences, and discrimination (1). From the perspective of rights and universal health coverage, there is a need to develop policies that consider this underserved population, and to include them in the health systems of the recipient countries (2, 3). Access to health care in the context of migration is one of the structural determinants of the health outcomes of migrants (4).

Access to health services is defined as the possibility a person has of getting in contact with a service when in need, and to see the need solved -within the ability of current knowledge and procedures- (5–7). The classic concept of access by (7) included five dimensions: i) availability (the presence of health care facilities); ii) accessibility (geographical proximity); iii) accommodation (the fit between the organization of services and the characteristics of potential users); iv) affordability (related to cost); and v) acceptability (perceptions of potential users regarding the services, as well as the attitudes of providers toward users). Later models have kept similar dimensions, adding the role of demand-side (potential users) characteristics to the supply-side (health system) ones in determining the possibility to access care.

More recently, (6) proposed a model centered on the person (or “patient”), in which access is understood as a process that begins with a health need, and in the best case scenario continues through the contact and interaction with health services, ending in the resolution of the need. The model emphasizes the interplay between people's “abilities” and the characteristics of the health services, and how elements from both can impact access. A recent scoping review found that Levesque et al.'s framework has been increasingly employed in the past few years, because of its combination of individual and health system factors, and also for its focus in access as a process with multiple steps where barriers can occur (8). However, only nine of 31 studies included in that review were conducted in low- and middle-income countries (LMICs), and only one of them (9) focused of migrants. Another six studies which did focus on migrants had been conducted in high-income countries of Europe or Eastern and South Eastern Asia. To the best of our knowledge, this is the first studies to apply Levesque et al.'s framework to the health care access of migrants in Latin America.

Historically, the main migration flow in Mexico has been the movement of Mexicans to and from the United States of America (US). Added to this, Mexico has been a major route for in-transit migrants from other countries who aim to reach the US. More recently, the number of in-transit migrants has increased, their demographic profile has diversified, and this flow has included more and more persons who flee their countries of origin because of violence, natural disasters, or political prosecution, and intend to apply for asylum (10). Therefore, what used to be a population of in-transit, mainly economic migrants, is better described now as a mixed migrant flow, composed of economic migrants, asylum seekers and displaced persons (11).

In 2018, during the Trump administration, the US federal Government implemented the “Remain in Mexico” program, which forced asylum seekers presenting at the Mexico-US land border to wait in the former country while their case was being considered in the latter (12). Following the onset of the COVID-19 pandemic, a second obstacle was passed by the US Government. This was the “Title 42” measures, which allowed migration authorities to return persons to Mexico without processing their asylum claims, on the basis of the health risks associated with receiving them in migration facilities (13). This combination of migration policies has resulted in large numbers of migrants becoming stranded in Mexican border cities for prolonged periods, many of them residing in migrant shelters operated by civil society organizations (CSOs). Although there are no accurate estimates of the number of migrants living in these conditions, some studies show that the arrivals into Mexican border cities of persons who aim to apply for asylum in the US have been in the thousands per month in the past years for cities such as Ciudad Juárez and Tijuana, with occasional outbursts such as the “migrant caravans” (14). In the fiscal year 2021, there were 1.7 million encounters with (persons detained by) the US Border Patrol in the US-Mexico border (15), and even though this number includes an important percentage of recidivists, it still points to the important numbers of persons that can at some point be staying in cities in the Mexican side of the border.

In this new situation, the Mexican health system has the challenge of guaranteeing migrants access to health services, as mandated by its recognition of health as a human right independent of migration status (16, 17). A recent amendment to the Art 77 bis 7 of Mexico's General Health Law grants free access to public health services (including medicines and supplies) to all persons in the country, so that migrants (including irregular migrants) are entitled to health care at all levels of the health system (18). However, an implementation gap remains, and in practice the members of mixed migrant flows are not always able to access services (19).

Our objective in this article is to analyze the barriers to access the public Mexican health system, encountered by migrants in cities in Mexican states at the Mexico-US border during the COVID-19 pandemic. We employ Levesque et al.'s model as an analytic tool to describe how supply- and demand-side elements interact in the process of access. In what follows, we begin with a brief description of Levesque et al.'s model. Then, we present the methods and results of our study. We close with a discussion of the main barriers faced by migrants, and a reflection on Levesque et al.'s model.

### Levesque et al.'s Health Access Model

As referred above, (6) describe access as a process that goes from health care needs to health care results. At each phase of the process, a person's abilities interact with dimensions of the health care system in a way that either hinders or facilitates access. The five abilities, according to the framework, are: 1) to perceive a health need; 2) to seek care; 3) to reach health care services; 4) to pay for services; and 5) to engage with health care. These abilities are determined by personal characteristics, as well as by the social context at different levels. Correspondingly, the five dimensions of the health care system are 1) approachability by potential users; 2) acceptability by potential users; 3) availability and accommodation to the potential users' needs; 4) affordability for the users; and 5) appropriateness of the service.

The process begins with the ability to perceive a health need, and to know which health care is required and exists. This perception can be determined by a person's knowledge and beliefs related to health. The system's approachability also influences those perceptions, so that people in need of care are either detected by the system (*via* screening) or know that the system is there for them to use. A health service can be more or less approachable as it becomes more or less visible to potential users, for example by means of outreach strategies.

In the second phase, access depends on a person's ability to seek care in a given service, a decision that will depend on personal values, cultural norms and the knowledge of health care options. Ability to seek is related to personal autonomy. It also depends on the acceptability of the system to the people it is supposed to serve. A service will achieve acceptability if its professional values, norms, and culture are adjusted to those of potential users. If services are less acceptable to some groups of the community, then inequities in access will result. For example, discrimination of some groups can make a service less acceptable to ethnic, sexual or other minorities.

Thirdly, a person needs to have the ability to reach the system, getting in actual contact with it. Among the personal characteristics that can limit the ability to reach are difficulties with physical mobility, working hours or living arrangements. Also important in this sense are material resources (e.g. economic means for transportation). On the supply side, geographical location, hours of opening and facility settings are examples of characteristics that constitute the dimension of availability and accommodation. Administrative or bureaucratic barriers can also be included in this dimension.

Fourthly, ability to pay is an important determinant of access, and is dependent on income and other sources of economic resources such as savings, and loans, that allow the person to cover health care expenditures, ideally without incurring in catastrophic expenses. It also encompasses the opportunity cost related to loss of income when an individual dedicates time to health care seeking. The relevance of individual ability to pay, however, is contingent to the health system's organization in terms of its charging (or not) for services, out-ot-pocket expenses vs. different types of insurance, cost of services, and social insurance schemes, which define the system's affordability dimension.

Finally, the ability to engage with the system once contact has been established requires all the elements that enable a person to adhere and follow up with treatment. Ability to engage refers to individuals' participation and involvement in their health-related decision-making including treatment. On the supply side, this requires an organization capable of providing continuity of care, and good quality services that can address the health need, which are part of the dimension of appropriateness. Appropriateness is thus the fit between services and the clients' needs. The appropriateness of a service is determined by the correct assessment of a health problem and quality of treatment (both technical and interpersonal).

## Materials and Methods

### Design

In this article, we report on the secondary analysis of data from a comparative case study (20). The parent study's objective was to analyze how migrant shelters acted during the COVID-19 pandemic in order to promote migrants' right to health. This was a mixed methods study, in which quantitative and qualitative methods (described below) were combined with a complementarity rationale, meaning the methods were used to measure overlapping but also different facets of a phenomenon, yielding an enriched, elaborated understanding of it (21). In the study design, the two methods had the same importance, and were implemented simultaneously and interactively. Four shelters (cases) were selected for the parent study, with the aim of obtaining a sample that varied in terms of internal capacities (as evidenced by time in operation, internal organization and services that the shelters provide beyond humanitarian assistance, number of paid staff, and number of people they could receive) and context (presence in the city of other migrant protection organizations, presence of federal human right agencies, population of the city, number of health care facilities and personnel). Data were collected from June to September 2021.

The quantitative component consisted of a survey of migrants residing in or receiving services such as food donations from the shelters. Since the main focus was on health care access, a non-probability, theory driven sample strategy was followed, in which we recruited participants who had experienced a health need during their time in the shelter. Given budgetary and logistic limitations, we did not calculate a sample size, but aimed to reach a quota of 80 adults who had experienced a health need during their time in the shelter, 80 adults in charge of a minor who had experienced a health need during their time in the shelter, and 80 women who were pregnant or had recently given birth. For the purposes of this article, we employ the survey's responses of the first two groups, and of women who reported that, in addition to their pregnancy-related need, they had experienced another health issue during their time in the shelter. Selection criteria for the quantitative phase were: a) having stayed or being in touch with the shelter for at least two nights; b) being 18 years of age or older; and c) having had a health need (or being in charge of a minor who had one) during their time in the shelter. The survey included questions about sociodemographics, migration, health issues, health care seeking, health services utilization, and satisfaction with health services.

The qualitative component consisted of semi-structured interviews with key actors of the response to migrants' needs. This included staff and volunteers of the migrant shelters, and informants working with SCOs, governmental and international agencies. Selection criteria for this component were: a) having knowledge of the situation of health and health care access of migrants in the city; and b) being 18 years of age or older. The interview guide was designed to target the parent study's objectives of describing the elements that facilitated or hindered the shelters' health-related response. It included questions on the main health care needs of migrants from the informants' point of view, barriers and facilitators to health care access, and how the shelters responded to the above before and during the COVID-19 pandemic. Since data collection took place during periods of high transmission of SARS-CoV-2, we conducted all interviews over video-conference. We recorded the audio of the interviews, and, because of budgetary constraints, conducted analysis of the records without transcribing them. To avoid losing important aspects of the data, we prepared a matrix with the main analytic dimensions of interest, and two researchers independently listened to the records and took notes about each dimension, as well as on novel aspects emerging from the interviews. When a quote was considered of relevance, the researcher transcribed it into the matrix. Then, a round of discussion was conducted between all researchers, in which the matrix was refined. The main qualitative data source for the analysis in this article was the final version of the matrix, but we went back to the original recordings for reference when needed during the iterative process of discussion of results described below.

### Analysis

For this article, we applied Levesque et al.'s model a posteriori (i. e., we did not use it as a guide for study design). Instead, after data collection we identified that our results regarding migrants' health care access could be best summarized by this model, and conducted a secondary analysis integrating quantitative and qualitative data with the model as a framework. In accordance to the mixed methods approach of the study, we sought to increase the meaningfulness and trustworthiness of the results by illustrating them with results from both methods.

In order to do this, we followed an iterative cycle, in which all authors of this article equally participated. First, we familiarized ourselves with the matrix of interviews results and with the quantitative results. Second, we prepared a table with the five abilities and dimensions proposed in the model, and classified the results according to them. We then discussed how well the results mapped to the dimensions and abilities, and changed the table's content accordingly. We also took notes on aspects of the results that did not match the model. Finally, we employed the final version of the table to organize the presentation of results according to each phase of the process in Levesque et al.'s model, considering both demand side (abilities) and supply side (dimensions) elements at each phase.

### Ethical Considerations

The study protocol was reviewed and approved by the Ethics Committee of El Colegio de la Frontera Norte. All participants were informed of the objectives and procedures of the study, or their right to refuse and withdraw consent, and of the measures that would be taken to preserve confidentiality. Participants read or were read an informed consent script, and gave verbal consent.

## Results

We recruited 219 migrants for the quantitative survey. Of them, 189 had valid responses to questions regarding health care seeking, and are therefore included in this analysis. Their distribution by groups and general characteristics of the analysis sample appear in Table 1. Most of them were originally from Central American countries, had a low level of education, and were waiting to cross to the US or had been returned after crossing.

**Table 1:** Characteristics of migrants surveyed in shelters (*n* = 189)^a^, by group.

**Variable**	**Adults with a health care need** **(*n* = 102)**	**Minors with a health care need^**b**^** **(*n* = 74)**	**Women with a pregnancy-related need** **(*n* = 13)**
Female	32 (31%)	33 (45%)	13 (100%)
Age, years (mean, s.d.)	36 (15)	5 (4)	24 (4)
**Country/region of birth**			
Central America Mexico Colombia Cuba	90(88%) 10 (10%) 1 (1%) 1 (1%)	62 (84%) 12 (16%)	11 (85%) 2 (15%)
Years of education (mean, s.d.)	8 (3)	4 (2)	6 (2)
Migration plans			
Returned from the US, plans to cross again^c^	22 (22%)	21 (28%)	2 (15%)
Waiting to cross into the US	63 (62%)	49 (66%)	11 (85%)
Plans to stay in Mexico or return to country of origin	17 (17%)	7 (9%)	

a *Analysis sample: participants who responded questions on health care seeking*.

b *As reported by the adult in charge of the minor. Migration plans in this group refers to plans that include the minor, and responses are not mutually exclusive*.

c *Returned as part of the “Remain in Mexico” policy, expedited return or deportation*.

For the qualitative component, we interviewed 22 key informants, of whom 11 were women and 11 men. Two were academics (both of also active in CSOs), nine worked for international organizations, nine for CSOs, and two for governmental agencies (one in health services, one in migration).

As per our sampling strategy, all migrants (or a minor they were on charge of) had experienced a health issue during their time in the city. The most frequently reported issues among adults were acute respiratory infections (including COVID suspect or confirmed cases), injuries as a result of accidents or violence, and mental health issues (self-reported depression, anxiety and insomnia). Likewise, acute respiratory infections were the most frequent health need among minors, followed by headache with non-specific causes, allergies and asthma.

**Figure 1:**
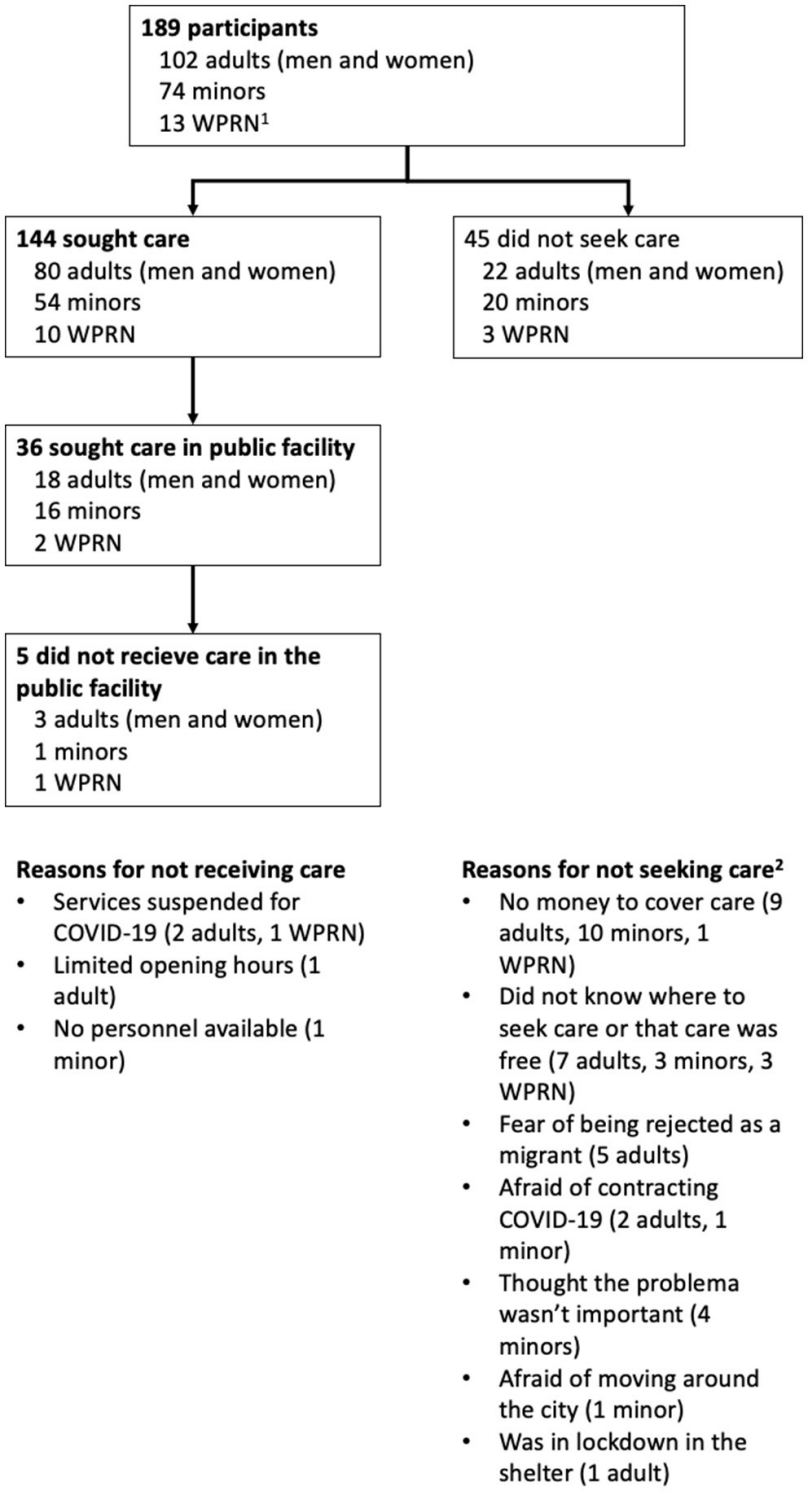
Health care seeking by migrants who responded the quantitative survey. ^1^WPRN: Women with a pregnancy-related need, responding questions on an additional, unrelated health need. ^2^Responses to this question were not mutually exclusive. For minors, the reason was given by the adult in charge of them.

Figure 1 shows the number of participants with valid responses in the questions about health care seeking. Of the 144 who sought care, 36 did so in a public facility. The rest of them were mostly seen in the shelters, in pharmacies, or in private services. A small number (7 cases) employed non-medical services such as a traditional healer, acupuncture or another.

### Step 1: System's Approachability and the Person's Ability to Perceive

In order to be approachable, a health system must reach out to potential users. According to interviewees, the public health system sometimes engages in outreach activities in the form of visits to the shelters of teams from the local health jurisdictions that provide preventive services and care. In one of the cases, a doctor had been commissioned by the jurisdiction exclusively to provide services to migrants. Most of these services had been scaled back in the first months of the COVID-19 pandemic, but at the same time the health jurisdiction got in touch with the shelters' director in order to facilitate detection and care of COVID-19 cases.

There's a [primary care clinic] close to the shelter. Since I've been here they [periodically] invited us three or five times to receive them for health fairs […] [IN THE FAIRS CONDUCTED IN THE SHELTER] they provide vaccination, talks, and other activities (*Female, staff of shelter*).

Approachability also requires that the system provides persons with information about available health care options, but the outreach activities described did not include this component. Added to this, we found that not knowing where to seek care was the second main reason for leaving a health need unattended, as reported by 12/45 (27%) participants who had not sought care. This was also mentioned by key informants.

[MIGRANTS] are not going to go to the health services because they do not have any type of advice or anyone to guide them or refer them to where to go in case they require attention (*Female, staff of international organization*).

While we did not collect detailed information on the ability to perceive a health issue as a need, according to the survey only in four cases (all of them of minors) care was not sought because the informant didn't think the problem was important. Two of these had symptoms of an acute infectious disease, one had chickenpox (as reported by the adult), and the other one's only symptom was lack of appetite. None of the adults abstained from seeking care because of this reason, so it seems that the need for health care is perceived by participants, but barriers arise in other aspects.

Thus, the main barrier to access in this step that we identified was lack of information about the services available, an aspect that was not considered in the health jurisdictions outreach activities.

### Step 2: System's Acceptability and the Person's Ability to Seek

Acceptability, in the sense of conflict between the health care system organization and migrants' values or cultural expectations, did not appear as an issue in either the quantitative nor the qualitative components of our research. None of our interviewees mentioned cultural aspects that could impact the public health system's acceptability to migrants, and neither did respondents to the survey cited this as a reason for not seeking care. However, some mentioned that lack of awareness of migrants' rights on the part of health system workers was a barrier to access.

[…] we have challenges associated with the lack of knowledge, as I was telling you, of the rights and the possibility of accessing, the rights of persons, because of lack of knowledge on the part of […] health system staff (*Female, staff of shelter*).

Since 45/189 (24%) of participants in the survey had not sought care, there was evidence of limitations in the ability to seek. The main reason for not seeking care they referred was lack of money to pay for it, mentioned by 20/45 (44%), so that ability to pay and affordability, which are part of the fourth step in the model, actually had effects earlier in the process, by limiting a person's ability to seek care.

Another barrier to the ability to seek was migrants' lack of awareness of their right to receive care in the public services, which was mentioned by key informants during qualitative interviews.

[…] many of them, not being familiar with the city, didn't know that they are supposed to be accepted in [public health care services] (*Female, staff of international organization*).

Thus, the main limitations in this step were lack of awareness of migrants' rights on the part of health care staff (which could be considered an element of the professional culture), and lack of ability to pay (which will be described in more detail in step four below).

### Step 3: System's Availability and Accommodation, and the Person's Ability to Reach

In the cities where the four shelters were located, public health services were geographically available. All four had primary care services within a five-kilometer radius (an indicator of geographical accessibility) (22). Three of them also had general hospitals within that distance, and one a maternity hospital in that range. All four cities had general hospitals, and three had specialty hospitals (e.g., geriatrics, children's hospital). Distance from a healthcare facility was not listed as an issue among the migrants who had not sought care when experiencing a health issue.

However, location is not the only aspect of geographical availability that matters for access (8). The time and cost of traveling to the health care facility are also important in this regard, and, as one interviewee pointed out, the cities where the shelters are located have deficient and costly public transport, and poor walkability. Extreme climate and security (e.g., safety, crime) concerns, as well as lack of knowledge of the surroundings, also constituted barriers for migrants' ability to reach care in public health facilities.

It's a challenge, because […] all health services are either there [DOWNTOWN] or in another area, which is [NAME OF ZONE] […] And that is the middle-class, more established area, so the shelters are far away (*Male, staff of international organization*). Added to that, another challenge is transport, [NAME OF CITY] is a city made for cars, public transport is deficient, apps like Didi or Uber are too expensive (*Female, staff of international organization*).

According to most interviewees, a major barrier in accessing the public health system was the exigency of presenting certain identification documents before being admitted for care in either primary care clinics or hospitals (this did not seem to be a barrier in the case of emergency services). This administrative barrier can be situated in the dimension of accommodation in the Levesque et al.'s model, as it implies a lack of adaptation of the system to the characteristics of its potential users (in this case, migrants without the required documents). While some interviewees mentioned that since the recent changes to the Mexican Health Law documents were no longer required by the public health facilities, most reported that this requisite was still in place. None of the migrants who responded the survey failed to receive care because of this reason, but it is possible that they were aware of the potential difficulty, and therefore did not even bother to go to the public services when in need. Access in these circumstances depended on discretionary decisions by the staff of public services, and of the arrangements migrant shelters were able to come to with them.

Migrants are required to present and ID, or a letter from the migrant shelter, in order to be seen at the General Hospital. They can't just go to any primary care facility. We send them to the [NAME OF PRIMARY CARE CENTER] which is 10 min away by car and 40 min away walking, because there they accept them with or without an ID. There are three primary care centers where migrants without papers can go, not all centers receive them. There's one more that does receive asylum seekers as they already have a CURP. Whether they are received without or with identity documents depends on the center's administration (*Female, staff of shelter*).

Therefore, while geographical availability in terms of location was not a major barrier in the cases we studied, there were other difficulties related to transportation, and administrative barriers affecting the migrants' ability to reach the system also emerged. Support by CSOs and international organizations was the main facilitator in this step, as they provided transportation, accompanied migrants to help them navigate the system, or, as in the quote above, made agreements with the public health services so they would receive migrants sent there from the shelters.

### Step 4: System's Affordability and the Person's Ability to Pay

Even though Mexico's public health care system is free of charge for all of those who have no other source of social security-related health services (18), chronic underfunding and other problems mean that, in most cases, patients have to pay out of pocket for expenses such as medicines or laboratory tests that are not available in the health care facility (Centro de Investigación Económica y Presupuestaria (23, 24). This constituted a major barrier, which as described above was the most frequent reason for not seeking care mentioned by the survey's respondents. Of the ones that sought and received care in the public system, 11/31 were required to pay for either medicines, laboratories or other services.

The migrants' ability to pay was limited by the fact that most of them had no source of income or were living of their savings. Besides, monthly income was low: 53/76 participants who responded a question about it had an income of <$3,500 Mexican pesos (about US$175), under the country's minimum wage (about US$220 at the time). Sometimes migrants were able to surpass the economic barriers to access public health services with the aid of the SCO's operating the migrant shelters.

The shelter covers the expenses and medicine required when the primary care center doesn't have the medicines, or when the migrant doesn't have the resources. If there's a medicine needed, we check in the shelter to see if we have it, and if we don't there's an agreement with a pharmacy. The medicines are paid with funds from UNHCR and with money that we receive as donations (*Female, staff of shelter*).

Another aspect of affordability and the ability to pay had to do with the costs of transportation. We have mentioned this before, as part of the ability to reach, but as other authors have pointed, transportation could also be considered part of the expenses a person has to incur in order to reach services (8).

Thus, affordability was one of the dimensions in which barriers to access were more apparent in our data. As with accommodation, it could also influence the migrants' perception of the system, so that their ability to perceive could have been diminished by knowing beforehand that they would not be able to pay for care, and therefore decided not even seek contact with the health care facility.

### System's Appropriateness and the Person's Ability to Engage

The dimension of appropriateness is better assessed through the results of the contact between the person and the health system, ideally ending in the resolution of the need. In our data, we only have evidence of the degree of satisfaction of those who had received some form of care in the public health system, which in general was high: 12/15 adults receiving care for themselves in the public system and 13/15 in charge of a minor who received care in the same system said they were satisfied with care. On the other hand, a process indicator that reflects the quality of care, and also the ability to engage on the patient's side, is whether the health care provider facilitates the patient's autonomy by explaining him/her the diagnosis and procedures to be followed. When asked about doctor-patient interactions, almost all who were seen in the public system or brought a minor in to receive care in it, reported they had been treated with respect, received a diagnosis, and were provided with written indications and explanations for how to take medicines. The only aspect relatively lacking was that 4/15 had not been asked if they had any doubts.

Thus, once in touch with the public health system, appropriateness and ability to engage seemed to be reasonably good. However, the small number of cases and lack of information on the resolution of the health need limits our capacity to reach conclusions in this regard.

## Discussion

**Figure 2:**
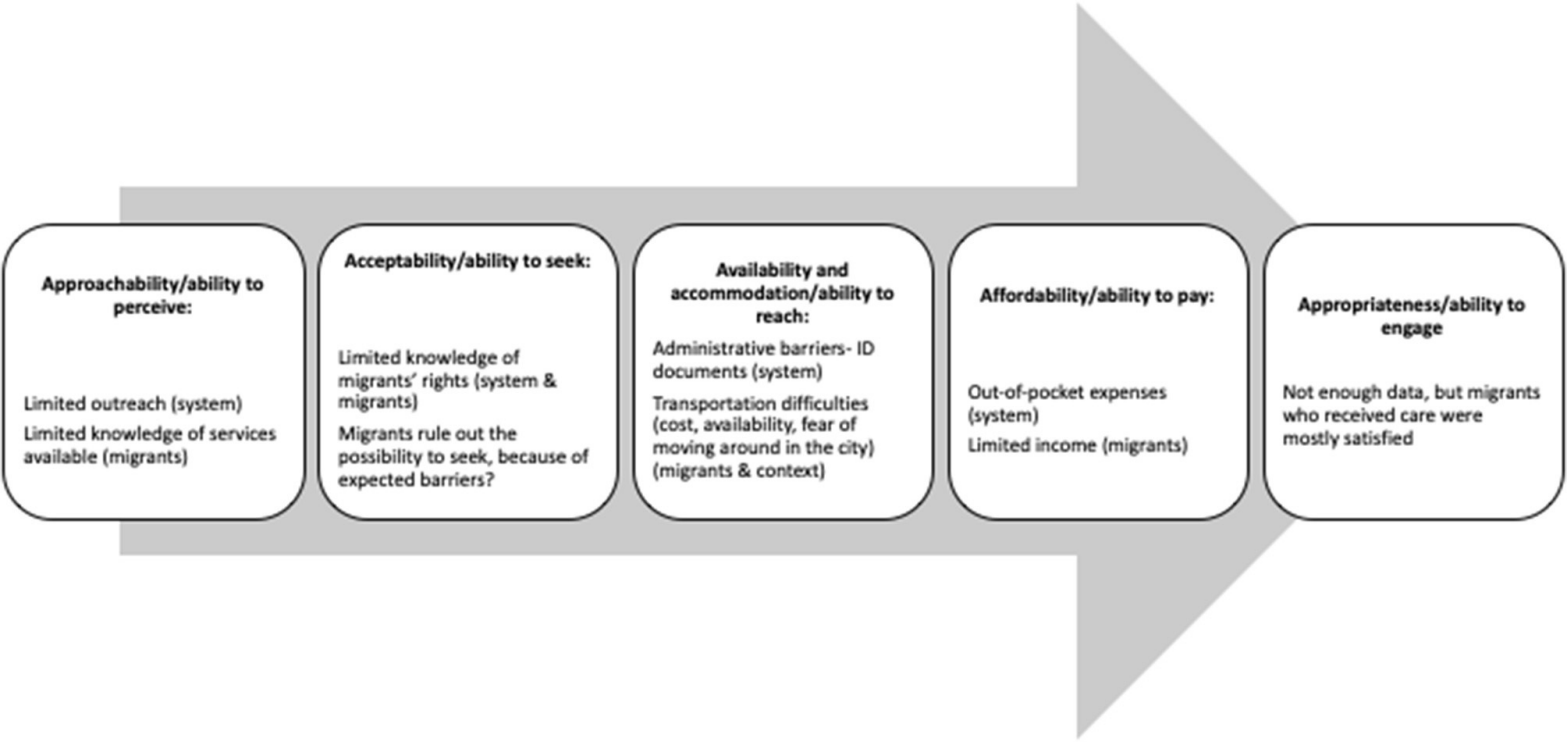
Barriers in the process of accessing health care. ^1^Adapted from (6).

In this article, we identified the barriers to access the public health care system in Mexico faced by members of mixed migrant flows. Given that according to the law the public system is open at all levels, free of charge, for migrants in Mexico, the fact that it is not the most sought-after option points to the fact that an implementation gap remains in Mexican health policies. We framed the barriers that help to explain this gap using Levesque et al.'s model. Our main conclusions are depicted in Figure 2.

In summary, we found that the main barriers occurred in the dimensions of affordability/ability to pay and availability and accommodation/ability to reach. This is similar to the results of other studies using Levesque et al.'s framework, which also have identified these two areas as relevant (8). An important finding, however, was that barriers classified as part of those dimensions could operate even in the first steps of the process, as migrants' ability to seek health care in the public health system might be hindered if the expectation of barriers makes them rule it out as a possibility. The decision to seek care in the public health system is also probably made after weighing the costs and benefits of different therapeutic options as well as the severity of health needs (25).

Using Levesque et al.'s framework in an empirical case study allowed us to assess its strengths and limitations. As other authors report, some results are difficult to categorize in a single dimension. For example, the distance or time to reach a health facility can be classified as an issue or availability, or of ability to pay (8). Some dimensions or aspects of dimensions that are important in the case of migrants are not completely captured by this model, as in the case of administrative barriers to access that persist even in the face of a normative right to care. Also, the model could be enhanced by considering the social determinants of individual abilities to access, such as the possibility of migrants to have decent work and sufficient salary, or their inclusion in other systems of social protection (1). Still, similar to other versions of access as a process (26–28), the model allowed us to systematically describe the barriers faced by migrants at each step.

A limitation of our study is that, since data came from a comparative case study of four sites, with non-probability sampling, we cannot claim that our results represent the situation of all member of mixed-migrant flows in Mexico. The intended sample size for women with reproductive care needs was not reached because there were not enough eligible women in the shelters during the period of data collection, and we had no members of the LGBTQ+ community among participants. Neither were we able to interview migrants who were not Spanish speakers.. Since not all shelters keep detailed records of the sociodemographic characteristics of migrants, we are not able to assess the representativeness of our sample, but as different groups might have different experiences in accessing health care, future studies should aim to include a more diverse sample. Another limitation is that data were collected during a peak of the COVID-19 pandemic, so some aspects of the health system's functioning might be different in comparison with other periods. However, the similarity of our results with some previous reports (29) makes us confident that they are a good representation of the situation. Not transcribing the interviews could represent a limitation, since transcription facilitates the management and sharing of information for analysis. However, in this work this limitation was resolved through the construction of an analytic matrix, and by having pairs of researchers listening to the interviews and filling the matrix, so that we were able to check the consistency of findings. Finally, we employed data from a study that was not designed with the Levesque et al.'s framework in mind.

As for the study's strengths, in contrast with others using this framework we were able to consider both the system's dimensions and the persons' abilities. Even if we did not have qualitative data on migrants' perspectives, we were able to include information from a migrants' survey, unlike some studies that only consider the opinion of health care providers and other experts.

To conclude, we found evidence that members of mixed migrant flows in Mexico experience barriers to access the public health care system, and identified the main dimensions in which those barriers appear. A corollary of our results is that legislation is not enough to ensure access, and there is a need to address the main gaps, removing administrative barriers, and ensuring that the public health system has the resources needed to protect its users from out-of-pocket expenses. Improving these aspects would be major steps in achieving the right to health for all, as mandated by the Mexican constitution.

## Data Availability Statement

The original contributions presented in the study are included in the article/supplementary material, further inquiries can be directed to the corresponding author.

## Ethics Statement

The studies involving human participants were reviewed and approved by Comité de Bioética, El Colegio de la Frontera Norte. Written informed consent for participation was not required for this study in accordance with the national legislation and the institutional requirements.

## Author Contributions

Conceptualization and writing—original draft preparation: CI and IB. Methodology: CI, IV-M, CR-C, GN, and IB. Analysis and writing—review and editing: CI, IV-M, CR-C, GN, SL-S, and IB. All authors contributed to the article and approved the submitted version.

## Funding

Funding for this research was provided by Hispanics in Philanthropy (HIP) (no grant number). The funding source had no role in the decision to publish, data analysis, or manuscript writing.

## Conflict of Interest

The authors declare that the research was conducted in the absence of any commercial or financial relationships that could be construed as a potential conflict of interest.

## Publisher's Note

All claims expressed in this article are solely those of the authors and do not necessarily represent those of their affiliated organizations, or those of the publisher, the editors and the reviewers. Any product that may be evaluated in this article, or claim that may be made by its manufacturer, is not guaranteed or endorsed by the publisher.
